# Multiple-Geographic-Scale Genetic Structure of Two Mangrove Tree Species: The Roles of Mating System, Hybridization, Limited Dispersal and Extrinsic Factors

**DOI:** 10.1371/journal.pone.0118710

**Published:** 2015-02-27

**Authors:** Gustavo M. Mori, Maria I. Zucchi, Anete P. Souza

**Affiliations:** 1 Center for Molecular Biology and Genetic Engineering, University of Campinas, CP, Campinas, São Paulo, Brazil; 2 São Paulo Agency for Agribusiness Technology, Piracicaba, São Paulo, Brazil; 3 Department of Plant Biology, Institute of Biology, University of Campinas, Campinas, São Paulo, Brazil; National Cheng-Kung University, TAIWAN

## Abstract

Mangrove plants comprise a unique group of organisms that grow within the intertidal zones of tropical and subtropical regions and whose distributions are influenced by both biotic and abiotic factors. To understand how these extrinsic and intrinsic processes influence a more fundamental level of the biological hierarchy of mangroves, we studied the genetic diversity of two Neotropical mangrove trees, *Avicenniagerminans* and *A*. *schaueriana*, using microsatellites markers. As reported for other sea-dispersed species, there was a strong differentiation between *A*. *germinans* and *A*. *schaueriana* populations sampled north and south of the northeastern extremity of South America, likely due to the influence of marine superficial currents. Moreover, we observed fine-scale genetic structures even when no obvious physical barriers were present, indicating pollen and propagule dispersal limitation, which could be explained by isolation-by-distance coupled with mating system differences. We report the first evidence of ongoing hybridization between *Avicennia* species and that these hybrids are fertile, although this interspecific crossing has not contributed to an increase in the genetic diversity the populations where *A*. *germinans* and *A*. *schaueriana* hybridize. These findings highlight the complex interplay between intrinsic and extrinsic factors that shape the distribution of the genetic diversity in these sea-dispersed colonizer species.

## Introduction

Mangrove plants encompass a polyphyletic and heterogeneous group defined by ecological and physiological traits that are adaptations to life within the intertidal zones of tropical and subtropical regions. These plants establish discrete communities, known as mangrove forests [1], which are globally distributed, covering approximately 137,700 km^2^ worldwide [2]. Within this larger distribution, mangrove species richness is heterogeneously distributed, such that the Eastern or Indo-West Pacific (IWP) region is one order of magnitude more diverse than the Western or Atlantic, Caribbean and East Pacific (ACEP) region [3]. Moreover, within each of these biogeographic regions, species number decreases as the latitude increases [3,4].

These patterns of species diversity are influenced by abiotic factors, such as oceanography, climate, topography and soil conditions [3]. Additionally, biotic factors play important roles in the maintenance of these patterns. For instance, the limited effective dispersal [3] and establishment of floating, long-lived, salt-tolerant propagules—with variable degrees of “viviparity” and continuous embryonic development without dormancy [5,6]—can affect species composition at many scales [3]. These issues concerning the distribution of mangrove species richness raise further questions regarding other levels of biological hierarchy, such as the organization of genetic diversity.

Since the 1980s, the genetic variation of mangrove plants at the molecular level has been evaluated using a variety of methods in both biogeographic regions [7]. More recently, a wide range of questions have been answered, and new patterns have emerged [8–12]. For instance, it is now clear that large land and ocean barriers to propagule dispersal are important evolutionary factors that differentiate mangrove populations [8]. However, at smaller geographic scales, substantial genetic structure is also observed among different genera [10,13]. Another important recent advance is that ancient and ongoing interspecific hybridization has now been recorded [8,11,14], including between taxa for which no morphologically intermediate individuals have been found [15].

**Fig 1:**
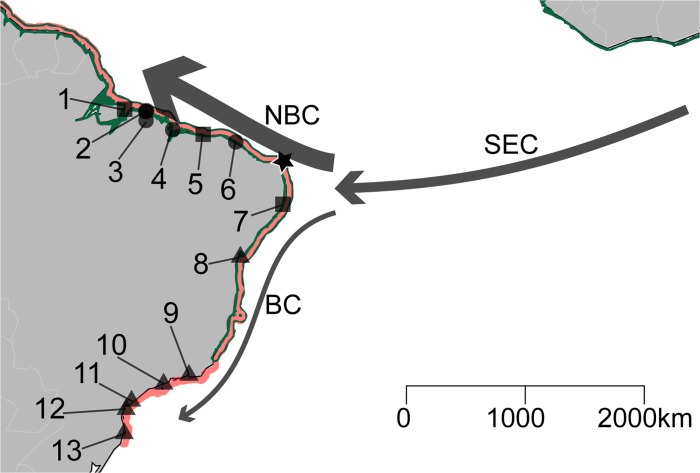
Geographic distribution of *A*. *germinans* and *A*. *schaueriana* samples along the Brazilian coast. A map of the South Atlantic showing the geographic distribution of *A*. *schaueriana* (red) and its sympatry region with *A*. *germinans* (green and red). Squares and triangles indicate locations where only *A*. *germinans* and or *A*. *schaueriana*, respectively, presented reproductive branches during sampling. Circles represent locales where both species presented flowers. The star indicates the northeastern extremity of South America. Sampling locations are displayed according to Table 1. Arrows denote the following near-surface ocean currents that influence the sampling range: the South-Equatorial (SEC), North Brazil (NBC) and Brazil currents (BC). Arrow sizes and line widths illustrate the mean current speed [61].

Using two Neotropical species of *Avicennia* L. (Acanthaceae) as models, we evaluated the extrinsic and intrinsic factors that shape the genetic variation of different species of mangrove plants. *Avicennia*, the most widely distributed genus of mangrove plants [16], is found in both the IWP (five species) and the ACEP (three species) regions. The ACEP *Avicennia* species with the largest distribution is *A*. *germinans* L., which is distributed across the entire region except for southern Brazil, where *A*. *schaueriana* Stapf and Leechman ex Moldenke are dominant (Fig. 1). These species are partially sympatric from the northeastern coast of Brazil to the northern limit of distribution for *A*. *schaueriana* in the lower Lesser Antilles [1,16]. Preliminary evidence of chloroplast capture between these species has been found within their sympatry zone [15]. This interaction is biologically feasible as both these species present a generalist pollination system and share pollinators [17,18]. Additionally, the reproductive phenology of these species overlaps in certain localities [19].

**Table 1:** Locality descriptions for the samples of *Avicennia germinans* and *A*. *schaueriana* from the Brazilian coast.

As	Ag	Locality (City, State)	Geographic Coordinate	Location in Fig. 1
	AgMRJ (31)	Soure, Pará	0° 43' 26" S, 48° 29' 24" W	1
AsSAL (22)		Salinópolis, Pará	0° 36' 36" S, 47° 22' 41" W	2
AsAJU (46)^$^		Bragança, Pará	0° 49' 12" S, 46° 36' 56" W	2^$^
AsPRM (47)		Bragança, Pará	0° 57' 42" S, 46° 37' 5" W	2
	AgPAa (28)*	Bragança, Pará	0° 54' 17" S, 46° 41' 15" W	2
	AgPAb (27)	Bragança, Pará	0° 56' 21" S, 46° 43' 17" W	3
AsALC (30)	AgALC (29)	Alcântara, Maranhão	2° 24' 37" S, 44° 24' 22" W	4
	AgPNB (29)	Parnaíba, Piauí	2° 46' 42" S, 41° 49' 20" W	5
AsPRC (31)	AgPRC (5)	Paracuru, Ceará	3° 24' 47" S, 39° 3' 23" W	6
	AgTMD (32)	Tamandaré, Pernambuco	8° 31' 35" S, 35° 0' 48" W	7
AsVER (31)		Vera Cruz, Bahia	12° 59' 1" S, 38° 41' 5" W	8
AsGPM (35)		Guapimirim, Rio de Janeiro	22° 42' 5" S, 43° 0' 26" W	9
AsUBA (32)		Ubatuba, São Paulo	23° 29' 22" S, 45° 9' 52" W	10
AsCNN (32)		Cananéia, São Paulo	25° 1' 12" S, 47° 55' 5" W	11
AsPPR (28)		Pontal do Paraná, Paraná	25° 34' 30" S, 48° 21' 9" W	12
AsFLN (66)		Florianópolis, Santa Catarina	27° 34' 37" S, 48° 31' 8" W	13

Sampled populations of *A*. *germinans* (Ag) and *A*. *schaueriana* (As) are indicated by three capital letters. Sample sizes are indicated with parentheses. The city and state in Brazil, geographic coordinate and numbers corresponding to Fig. 1 are denoted for each site.

* indicates a sample under a reduced inundation frequency [19];

^$^ indicates the locality where the progeny array was sampled.

We hypothesized that multiple geographic scales of genetic structure exist for *A*. *germinans* and *A*. *schaueriana*, as has been independently observed for many true and associate mangrove plants [8–10,20,21]. To evaluate this hypothesis and the potential intrinsic and extrinsic factors currently influencing these genetic structures, we characterized the population genetics of these species at three geographic scales, the *A*. *schaueriana* mating system, and hybridization between these species using microsatellite markers.

## Materials and Methods

### Plant material and sampling strategy

From June 2008 to December 2010, we sampled 400 individuals of *A*. *schaueriana* from 11 localities and 181 individuals of *A*. *germinans* from seven locales along the Brazilian coast, covering more than 4900 km of coastline, to evaluate local (thousands of meters, microscale), regional (hundreds of kilometers, mesoscale) and continental geographic scales (thousands of kilometers, macroscale) (Fig. 1, Table 1). Latitude and longitude were recorded using a global positioning system (Garmin 76CSx, WGS-84 standard, Garmin International Inc., Olathe, KS, USA). Licenses (17159 and 17130) to collect the leaves and reproductive branches of these species were obtained from the Instituto Brasileiro do Meio Ambiente e dos Recursos Naturais Renováveis (IBAMA, currently, Instituto Chico Mendes de Conservação da Biodiversidade, ICMBio).

Each species sample is presented in Table 1, with Ag and As indicating *A*. *germinans* and *A*. *schaueriana*, respectively; localities from which the samples were obtained are indicated with three letter abbreviations. For *A*. *germinans*, we collected individuals from two nearby localities in Bragança, Pará, Brazil that experience different tidal influences: one area in which inundation frequency was reduced due to changes in the hydrographic regime caused by highway construction (AgPAa) [19], and another that experienced a regular tidal pattern (AgPAb) (Fig. 1, Table 1). To minimize misidentification, we distinguished species in the field based on both vegetative and reproductive traits. We identified *A*. *germinans* individuals by their ovate leaves that usually present a blunt apex; by their long, exserted stamens; and by their conspicuously hairy petals. We identified *A*. *schaueriana* by their obovate leaves; by their inserted stamens; and by their glabrous inner-face corolla [1]. Voucher specimens from every location, except for the AsALC and AgALC samples, were deposited and cataloged in the University of Campinas (UEC) and Embrapa Amazônia Oriental (IAN) herbaria, both of which are located in Brazil. We sampled leaves from flowering trees located at least 20 m from any other tree, and leaves were stored in zip-lock bags containing silica gel. Leaf material was lyophilized and stored at −20°C prior to DNA isolation. Despite our best sampling efforts, we found few individuals (five) of *A*. *germinans* presenting flowers at AgPRC (Table 1).

In June 2008, we randomly chose 24 mother trees separated by at least 30 m from AsAJU for the *A*. *schaueriana* mating system analyses (Fig. 1, Table 1). In total, 288 healthy propagules attached to the tree were sampled from each of these trees, with a mean of 12 propagules per parental plant (ranging from 8 to 14). Each progeny was stored in an individual zip-lock bag in the field, and when brought to the laboratory, each fruit was stored in distilled water to isolate the pericarp from the embryo, which was stored at −80°C until DNA extraction.

### *A*. *schaueriana* microsatellite development and molecular biology procedures

For total genomic DNA isolation, we ground leaf samples and whole embryos into fine powder in liquid nitrogen, according to a cetyltrimethyl ammonium bromide protocol. The genetic diversity of each *Avicennia* species was analyzed using previously published microsatellite markers for *A*. *germinans* [22–24] and markers developed for *A*. *schaueriana* (described herein), which were isolated to achieve more reliable results using a larger number of loci for both species. The *A*. *schaueriana* microsatellites were developed using a method previously used to isolate *A*. *germinans* markers [24]. Monomorphic markers for *A*. *germinans* [24] were tested using a subsample of eight individuals from three and five locales for *A*. *germinans* and *A*. *schaueriana*, respectively. Markers showing intra- or interspecific polymorphisms were considered in subsequent examinations (Table 2).

**Table 2:** Microsatellites markers used in this study.

Marker	Primer sequence (5’-3’)	Size (bp)	GenBank	Repeat motif	AC	Reference	Ag	As	Both
Agerm1-02	F: TAACTAGCCGCCCATCCATC	168	HM470004	(ca)_11_	53.4°C	24	P	P	P
	R: ACCAGCCCACATCCAACAAT								
Agerm1-03	F: CCATGTTTTTGACTTTTTATTTTG	161	HM470005	(ca)_9_	48.2°C	24	P	P	P
	R: TTACGATAGGGTGGATTGAGATTTT								
Agerm1-06	F: GAATTGGCTGGAATGAGGAA	175	HM470008	(gt)_6_	63.4°C	24	P	P	P
	R: GTGTTTTGGAAGGAGCCTGT								
Agerm1-07	F: CCTGACACTCTGGGACATCA	157	HM470009	(gt)_9_	50.5°C	24	P	P	P
	R: CCTTTTGACGCATTTGTGG								
Agerm1-08	F: CTGCCGAGCAAAGGCTTA	183	HM470010	(tg)_9_	TD65-55	24	M	P	P
	R: GCAAGATCCACAGCTTCACA								
Agerm1-12	F: CAGTTTGGTGAGAAGGATGTT	127	HM470014	(ac)_15_	53.4°C	24	P	M	P
	R: TTTGAGGTCGGCTCGTTAAG								
Agerm1-14	F: CCAATTGTGTCGTCCTTTTA	159	HM470016	(ca)_8_(at)_6_	59.6°C	24	P	M	P
	R: AGCCTTACTTTTCCTTTGT								
Agerm1-15	F: ACTTACACACAAAATGCACA	248	HM470017	(ca)_4_. . .(ac)_13_	56.7°C	24	P	M	P
	R: CTGAGAGTGCCGACTGAATG								
Agerm1-16	F: CCTAATACAAATGACACTAAAA	176	HM470018	(tg)_9_	53.4°C	24	P	-	-
	R: TGCATGTCAATTATCAGTCT								
Agerm1-18*	F: CAGCGGGAAAAATCAAACCAA	243	HM470020	(ag)_16_	63°C	24	P	P	P
	R: CCTGTGCACATCCGCCTCTC								
Agerm1-21*	F: GGAGCAATTGTCGAAAGGAC	150	HM470023	(ca)_8_	61.8°C	24	P	-	-
	R: CGTTGCTGAGACAAGGAACA								
Agerm1-22	F: CACAGGTTCTACTCGGAAGATG	167	HM470024	(tttctt)_4_	63.4°C	24	P	M	P
	R: CGTCCGGGTCTACTCAAAAA								
Agerm1-25	F: GAGCAAAACTGGATACTCAAATG	237	HM470027	(tg)_10_. . .(tg)_4_	65.5°C	24	P	-	-
	R: AATAATAAGGCGCCCGTGT								
CTT01	F: CATCCACATTGCCCTGAT	102	DQ240228	(ctt)_8_	TD65-55	23	P	P	P
	R: GCCTGATAAGTTGAGTTGCTG								
T7*	F: CTAAGTAGGACAGTAATGCGAC	170	AY741799	(cat)_2_(at)_3_(gtat)_5_	48.2	22	P	P	P
	R: AATCATCAGAATCCCTCAAGTGC								
T8	F: ACACAACGCAGATAAATCC	112	AY741802	(tgta)_6_	59.6	22	P	^-^	-
	R: AATGATGCGCTGTCTCCGTC								
Aschau1-01	F: AACGACAAACCATTAGAAACCAA	219	KC783259	(tg)_21_. . .(at)_6_	46.7	This work	P	P	P
	R: CAATTGAATTTTCTGATTCCCTAA								
Aschau1-02	F: ACACTACACCCTTCAGCTCAATAA	150	KC783260	(ac)_15_	60	This work	P	P	P
	R: ACCCCCAATGGTAGGACAT								
Aschau1-03	F: GCGGTATCTCCCGTGATTT	227	KC783261	(ca)_9_gc(ca)_14_	60	This work	P	P	P
	R: TAGAGGGGAGATTGGTGTGG								
Aschau1-04	F: ACGTAAGCTGTGGACGAAGG	218	KC783262	(ct)_6(_ac)_6_gc(ac)_5_	60	This work	P	P	P
	R: AAGGGATGGGAAGTGGATTC								
Aschau1-05	F: TCTAATTGGACGATGGCAGA	179	KC783263	(gt)_4_gc(gt)_5_tt(gt)_4_	60	This work	P	P	P
	R: TGTAGCTGAAATTCCCCTTTTT								
Aschau1-06	F: AACGTTTTGCCTACACCCTCT	176	KC783264	(ca)_14_	60	This work	P	P	P
	R: GCAAGAACTATCGTTCCATCA								
Aschau1-07	F: TGGCAGATGTGTCTTCCTGA	209	KC783265	(tg)_11_	56.7	This work	^-^	P	-
	R: CCTCAGACTTGAATCAGCAGTG								
Aschau1-08	F: AATAATTAAGCATCCACTCG	174	KC783266	(gt)_14_…(gt)_6_	TD 65-55	This work	P	P	P
	R: TTTAACTTGATGAGGAACTTG								
Aschau1-09	F: TATCCCTTTGCATTGTTTGAGT	202	KC783267	(ca)_21_	60	This work	M	P	P
	R: TTTCAACTCAACTTCATCCT								

Characteristics of the microsatellite markers developed for *Avicennia germinans* previously developed [22; 23; 24] and for *A*. *schaueriana*, described herein. The expected size based on the clone fragment, GenBank accession number, repeat motif, optimal PCR amplification conditions (AC) are shown for each marker. TD65-55 indicates touchdown PCR with temperatures ranging from 65 to 55°C. P indicates polymorphic and M denotes monomorphic marker for *A*. *germinans* (Ag) and *A*. *schaueriana* (As).

* indicates evidence of null alleles for three or more samples.

Polymerase chain reactions (PCRs) were performed as previously described [24] or with certain modifications [22, 23] (Table 2). Amplification products were visualized via vertical electrophoresis in 1× TBE, 6% polyacrylamide denaturing gels stained with silver nitrate. Product sizes were determined by comparison to a 10 bp DNA ladder (Invitrogen, Carlsbad, CA, USA). Approximately 10% of the total samples were re-genotyped to evaluate scoring genotyping errors. To evaluate the *A*. *schaueriana* mating systems, only markers that presented polymorphisms at AsSAL, AsAJU and AsPRM were employed, except for the loci Agerm6 and Agerm8, out of a total of 13 markers.

### Intraspecific genetic diversity analyses

After characterizing the *A*. *schaueriana* markers, we tested the occurrence of linkage disequilibrium (LD) for all pairs of markers per sample using the FSTAT 2.9.3 software program [25]. As we found consistent evidence of LD for *A*. *germinans* and *A*. *schaueriana*, we considered 22 and 17 polymorphic markers for further analyses, respectively (Table 2).

Using the MICRO-CHECKER software program [26], we observed evidence of null alleles and stuttering (Table 2). However, despite these indications, we observed no substantial differences between the global and pairwise G_ST_ values of samples that were corrected or not corrected for null alleles using the “excluding null alleles” method implemented in the FreeNA software [27] (S1 and S2 Tables); therefore, we used the original dataset for further analyses. We evaluated intraspecific genetic variation based on the average effective number of alleles (N_e_), the expected (H_E_) and observed heterozygosities (H_O_), and the fixation index (f) for each sample using GenAlEx 6.5 software [28]. The apparent outcrossing rate (*t*_a_) was determined as (1 - *f*)/(1 + *f*) assuming Wright equilibrium [29] (Table 3). We tested each sample for Hardy-Weinberg equilibrium (HWE) using heterozygote deficiency as the alternative hypothesis in the Genepop 4.0 software program [30].

**Table 3:** Intraspecific genetic diversity of *A*. *germinans* and *A*. *schaueriana* samples from the Brazilian coast. Sample codes are denoted as in Table 1.

Sample	N_e_		H_O_		H_E_		f		t_a_
***A*. *germinans***									
AgMRJ	3.461	(0.559)	0.541	(0.056)	0.587	(0.05)	0.066	(0.049)	0.876
AgALC	2.731	(0.336)	0.452	(0.045)	0.540	(0.05)	0.132	(0.032)	0.767
AgPAa	2.356	(0.304)	0.437	(0.054)	0.452	(0.056)	-0.002	(0.029)	1.005
AgPAb	3.259	(0.506)	0.522	(0.056)	0.569	(0.053)	0.083	(0.048)	0.846
AgPNB	1.788	(0.170)	0.294	(0.046)	0.350	(0.053)	0.131	(0.036)	0.768
AgPRC	2.397	(0.179)	0.310	(0.049)	0.598	(0.048)	0.452	(0.079)	0.377
AgTMD	1.174	(0.064)	0.025	(0.012)	0.110	(0.037)	0.662	(0.083)	0.203
Average	2.452	(0.142)	0.369	(0.022)	0.458	(0.023)	0.174	(0.024)	0.704
***A*. *schaueriana***									
AsSAL	1.816	(0.247)	0.242	(0.050)	0.333	(0.066)	0.209	(0.056)	0.654
AsAJU	2.002	(0.349)	0.245	(0.051)	0.346	(0.066)	0.308	(0.075)	0.529
AsPRM	2.168	(0.443)	0.21	(0.043)	0.358	(0.071)	0.378	(0.04)	0.452
AsALC	1.879	(0.273)	0.255	(0.051)	0.336	(0.068)	0.207	(0.061)	0.656
AsPRC	1.279	(0.079)	0.136	(0.036)	0.178	(0.046)	0.217	(0.052)	0.644
AsVER	1.455	(0.170)	0.144	(0.036)	0.213	(0.058)	0.187	(0.073)	0.685
AsGUA	1.638	(0.319)	0.146	(0.041)	0.216	(0.065)	0.195	(0.055)	0.674
AsUBA	1.174	(0.080)	0.05	(0.022)	0.104	(0.044)	0.481	(0.095)	0.351
AsCNN	1.529	(0.202)	0.193	(0.060)	0.215	(0.067)	0.112	(0.077)	0.799
AsPPR	1.626	(0.321)	0.155	(0.049)	0.213	(0.067)	0.185	(0.049)	0.688
AsFLN	1.477	(0.211)	0.148	(0.052)	0.188	(0.064)	0.248	(0.072)	0.603
Average	1.64	(0.081)	0.175	(0.014)	0.246	(0.019)	0.242	(0.02)	0.610

Average effective number of alleles (N_e_), expected (H_E_) and observed (H_O_) heterozygosities and fixation index (f), with respective standard errors between parentheses, and outcrossing apparent rate (*t*_a_) denoted.

### Population structure within species

Considering the different sets of microsatellite markers for each species, we used different approaches to evaluate how genetic diversity within each species is organized. We assessed population structure using the summary statistics D [31] and G_ST_ [32] using the diveRsity package [33]. The reliability of these statistics was verified using 10^5^ permutations. We also verified the pairwise relations of these statistics between every pair of localities, and we evaluated isolation by distance (IBD) by performing a Mantel test in the ade4 software program [34], considering the approximate linear distance between the sample locales along the coastline and the summary statistics G_ST_ [32] and D [31]. The Mantel correlograms were calculated in the vegan package [35], considering 10 classes of geographic distances for *A*. *germinans* and 15 for *A*. *schaueriana*, with 10^5^ permutations performed to verify the significant correlations.

To further analyze how genetic diversity is structured within each species, we used a multivariate method known as discriminant analysis of principal components (DAPC) [36] implemented in the R package ADEGENET 1.3.7 [37], considering the samples from each locale as different groups. We applied this model-free approach, which provides principal components (PCs) that rely solely on the inter-population variability [36], and we retained seven and six PCs that represented 49.9% and 60.4% of the total genetic information of *A*. *germinans* and *A*. *schaueriana* samples, respectively, with the number of clusters (k) varying from 1 to 50. The number of PCs was chosen using the *optim*.*a*.*score* function to avoid over-fitting during discrimination. The choice of an optimal k value was based on the Bayesian information criterion provided for each k tested. We also used the model-based clustering method implemented in the Structure 2.3.4 software program, assuming correlated allele frequencies and admixture [38,39], and disregarding any *a priori* information. We performed 50 independent Markov chain Monte Carlo (MCMC) runs for each k, which ranged from 1 to 10 for *A*. *germinans* and from 1 to 15 for *A*. *schaueriana*, with 5×10^5^ iterations following a burn-in period of 5×10^5^ iterations. The k value that best explained our data was determined using both maximization of the logarithm likelihood of the data, lnL [38], and the *ad hoc* statistic ΔK [40]. We used the CLUMPP software [41] to address label switching and multimodality issues using the *Greedy* algorithm, with 10^6^ random input orders. For *A*. *schaueriana*, to determine fine-scale population structure revealed throughout the analyses, we used the same strategy described above for each of the two clusters we obtained (see below), with k ranging from 1 to 10 for each group. We used an extended model-based approach implemented in the InStruct software [42] to consider inbreeding—which is likely to be a violated condition in these species (see below)—without prior information regarding spatial location or sample membership. We performed five independent runs of 10^6^ MCMC repetitions with a 5×10^5^ burn-in period for each. We also performed hierarchical analyses of molecular variance (AMOVA) using permutation procedures (10^5^ iterations) in the Arlequin 3.5 program [43], considering the clusters identified using both multivariate and Bayesian methods. For this purpose, we considered the F_ST_ analog estimator under the infinite alleles mutation model (IAM) [44].

### Ongoing hybridization between *A*. *germinans* and *A*. *schaueriana*

To evaluate the ongoing hybridization between these *Avicennia* species, we used a different set of markers as only microsatellites that yielded PCR products for both species were considered to reduce marker development bias. We evaluated 20 microsatellites in these analyses, including intraspecific monomorphic markers that showed variation between species (Table 2).

The same methods used to study genetic diversity and population structure described above, with the exception of InStruct, were used in this investigation. For DAPC analysis, we used seven PCs, which explained 67.7% of the variance using every species sample as *a priori* clusters. In addition to using the same model-based clustering approach described above [38,39], with k ranging from 1 to 10, we arbitrarily defined a threshold of 0.15 for membership probability to assess any sign of historical hybridization. We also evaluated the existence and categories (F_1_, F_2_ and backcrosses between a “pure species” and a F_1_) of eventual two-generation hybrids between each “pure species,” employing a different model-based method implemented in the NewHybrids 1.1 beta software [45]. For this purpose, we evaluated the posterior distributions using five independent chains of 10^6^ MCMC following 5×10^5^ burn-in steps without prior allele frequency information, considering Jeffrey-type and uniform distribution priors for θ and π. We also evaluated the groups identified with these approaches using hierarchical AMOVA to evaluate how genetic diversity is organized between and within *A*. *germinans* and *A*. *schaueriana*.

### *A*. *schaueriana* mating system

Using 24 open-pollinated progeny arrays composed of 288 individuals, we evaluated the mating system of *A*. *schaueriana* under the mixed mating model for unlinked markers [46] using the program MLTR v3.4 [47]. According to this method, considering both Newton-Raphson (NR) and expectation-maximization (EM) methods regarding the absence and presence of null alleles, we estimated the multilocus (*t*_m_) and single-locus (*t*_s_) outcrossing rates, the outcrossing rate between related individuals (*t*_m_-*t*_s_), the average single locus inbreeding coefficient of maternal parents (F_m_) and the multilocus paternity correlation (*r*_p(m)_). Standard error was determined based on 10^4^ bootstraps among families.

## Ethics Statement

We confirm that we obtained two licenses (Nos. 17159 and 17130) to collect the leaves and propagules of *A*. *germinans* and *A*. *schaueriana* from the Brazilian Institute of the Environment and Natural Renewable Resources—IBAMA (currently Chico Mendes Institute for Biodiversity Conservation—ICMBio). We confirm that *Avicennia germinans* and *A*. *schaueriana* are not endangered or protected species.

## Results

### *A*. *schaueriana* microsatellite development and intraspecific genetic diversity

Of the 192 sequenced clones that constituted the constructed library, 52 presented 60 microsatellites. Based on these loci, we designed 43 primer pairs, 16 of which were excluded from further analyses due to amplification failure, unexpected fragment size or nonspecific products. Of the remaining markers, 18 were monomorphic for *A*. *germinans* and *A*. *schaueriana*, eight were polymorphic for *A*. *germinans*, and nine were informative for *A*. *schaueriana* (Table 2).

We observed varying degrees of polymorphism among markers within and between samples of each species in terms of N_e_, H_E_ and H_O_ (Table 3). We measured significant departures from HWE for every sample of both species. We also observed f values ranging from 0.112 to 0.481 for *A*. *schaueriana* and from −0.002 to 0.662 for *A*. *germinans*, with average values of 0.242 and 0.174, respectively. Therefore, there was evidence that these species exhibit mixed mating systems as the average t_a_ estimated for the former was 0.610 and 0.704 for the latter (Table 3).

### *A*. *schaueriana* mating system

Regardless of the method used (NR or EM, with or without considering the presence of null alleles), the results were similar and differed only when thousandths were considered; therefore, only the EM results disregarding null allele outcomes are shown. We observed a predominantly outcrossing mixed-mating system (*t*_m_ = 0.542 ± 0.062, t_s_ = 0.232 ± 0.065) for this progeny array, which was in accordance with the t_a_ (0.529) estimated for AsAJU, which was composed of only reproductive individuals, with F_m_ = 0.232 ± 0.065 assuming Wright’s equilibrium. We also observed that biparental inbreeding (*t*_m—_t_s_ = 0.151 ± 0.026) contributed to the apparent selfing rate of the progeny, which showed a fraction composed of full-sibs (r_p(m)_ = 0.178 ± 0.057).

### Population structure within species

**Fig 2:**
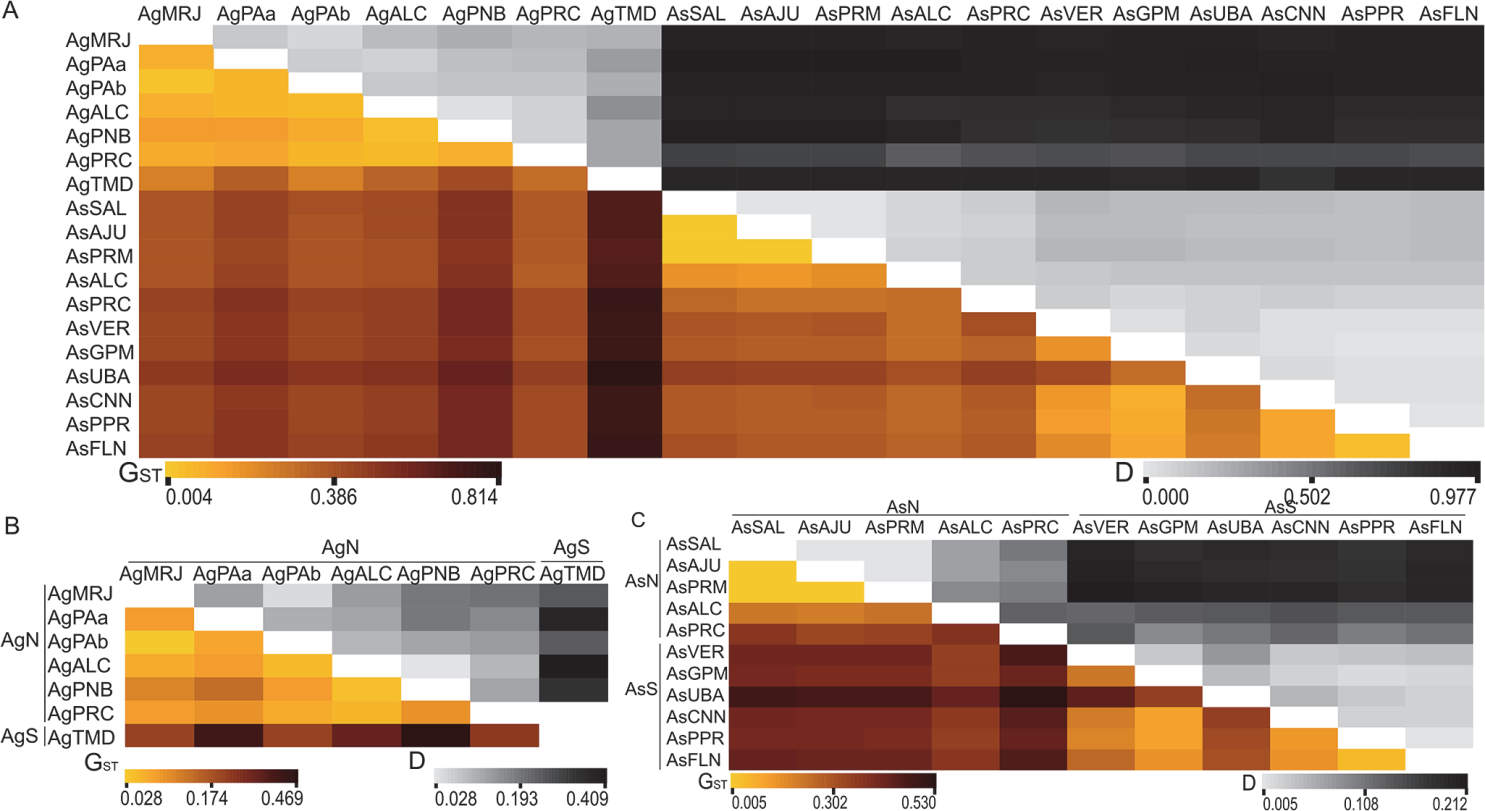
Pairwise comparisons of population genetic differentiation in *A*. *germinans* and *A*. *schaueriana*. Pairwise measurements of the genetic variation between samples in terms of Nei’s G_ST_ (1973—below diagonal) and D (Jost 2008—above diagonal) values in A) both *A*. *germinans* and *A*. *schaueriana*, B) *A*. *germinans*, and C) *A*. *schaueriana* samples. AgN and AgS and AsN and AsS refer to samples of *A*. *germinans* and *A*. *schaueriana* taken from locales north and south of the NEESA, respectively. All measures were significant (p < 0.05).

We found substantial evidence for differentiation among the samples with global G_ST_ and D values of 0.241 and 0.263 for *A*. *germinans* and 0.480 and 0.127 for *A*. *schaueriana*, respectively. The pairwise G_ST_ and D values for both species were highly correlated, indicating a varying degree of differentiation among different pairs of samples for both species (rho ≥ 9.11 for all cases, Fig. 2 and S3 Table), despite the lower values of D than G_ST_ for *A*. *schaueriana*. These results indicate that substantial genetic structure exists for both species, primarily when samples north and south of the northeastern extremity of South America (NEESA—Fig. 1) are considered (Fig. 2, S1 and S2 Figs.). For simplicity, we use “Ag” and “As” as acronyms for *A*. *germinans* and *A*. *schaueriana*, respectively, and “N” or “S” as codes for samples north and south of the NEESA, respectively.

**Fig 3:**
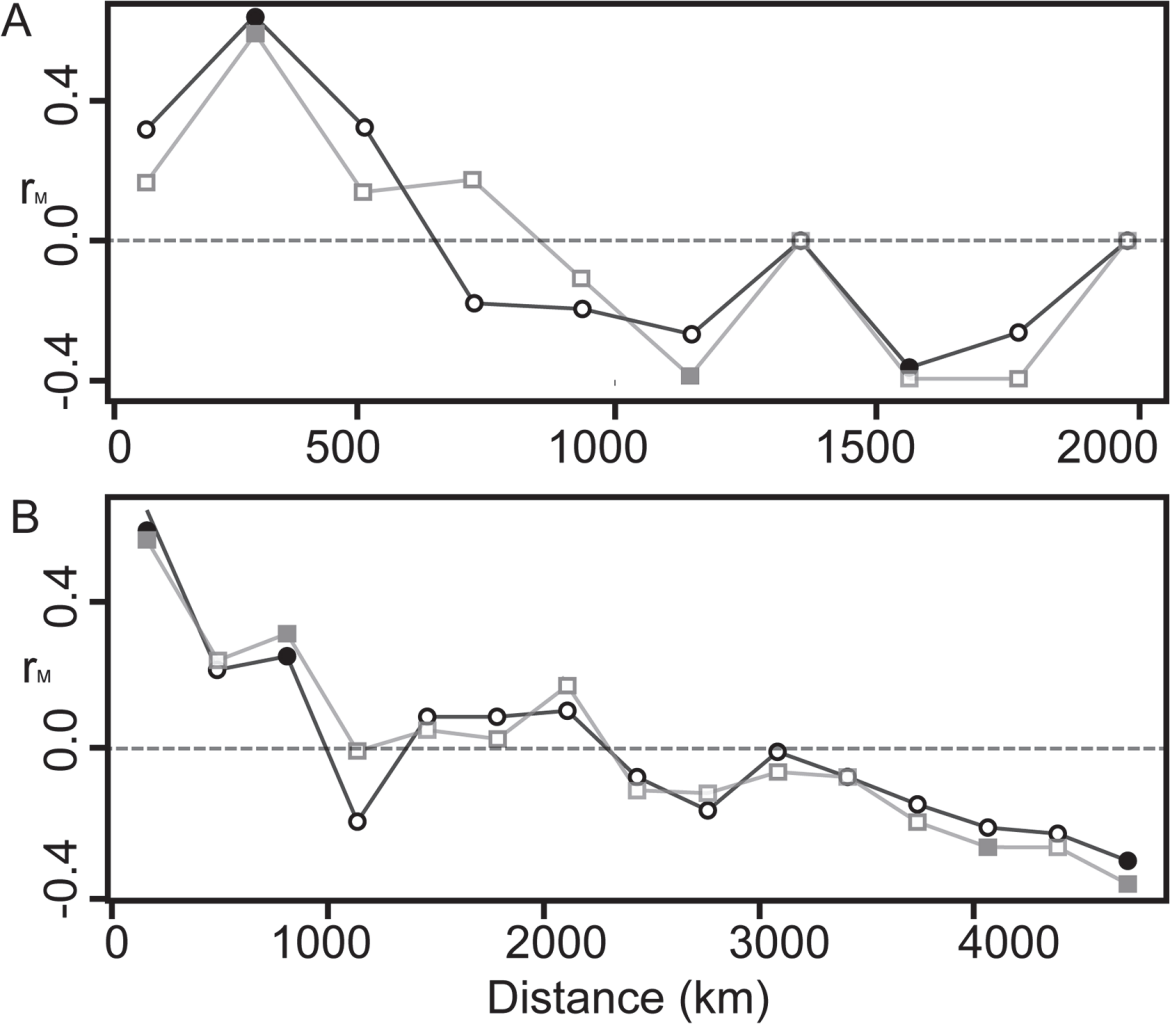
Isolation by distance of *A*. *germinans* and *A*. *schaueriana*. Mantel correlograms performed on approximated distances between each pair of sample locales along the coastline for A) *A*. *germinans* and B) *A*. *schaueriana*. Black interconnected circles refer to D [31], and gray interconnected squares indicate Nei’s genetic distance [69]. Filled squares and circles indicate significant correlations (p < 0.05) after 10,000 permutations.

**Fig 4:**
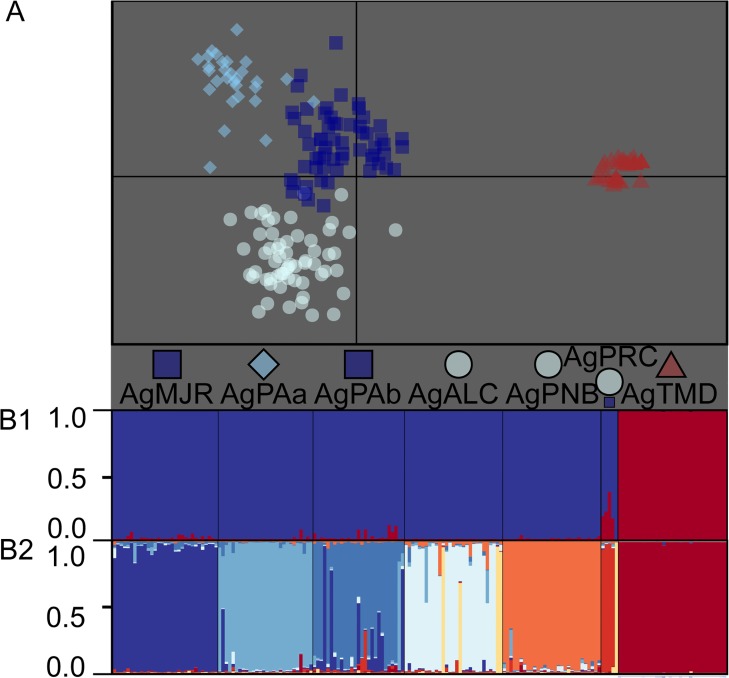
*A*. *germinans* population structure along the Brazilian coast. A) A scatterplot of the first two principal components of the multivariate analysis of DAPC [36], which resulted in a most likely k value of 4. Symbols indicate the group to which each individual was assigned. The geographic origin of each individual is denoted for each locality sample such that more than 10% of the total number of individuals was composed of the inferred cluster. A larger symbol indicates that a cluster was predominant in the locality sample (pairwise cluster ratio larger than 1:5), whereas equal symbol sizes indicate similar cluster contributions to the total number of individuals in each sample. B1) Model-based clustering analyses [38,39] considering k = 2 and B2) k = 8, where each individual is represented by a vertical line and each color refers to one inferred cluster; the posterior probability of group membership is indicated.

**Fig 5:**
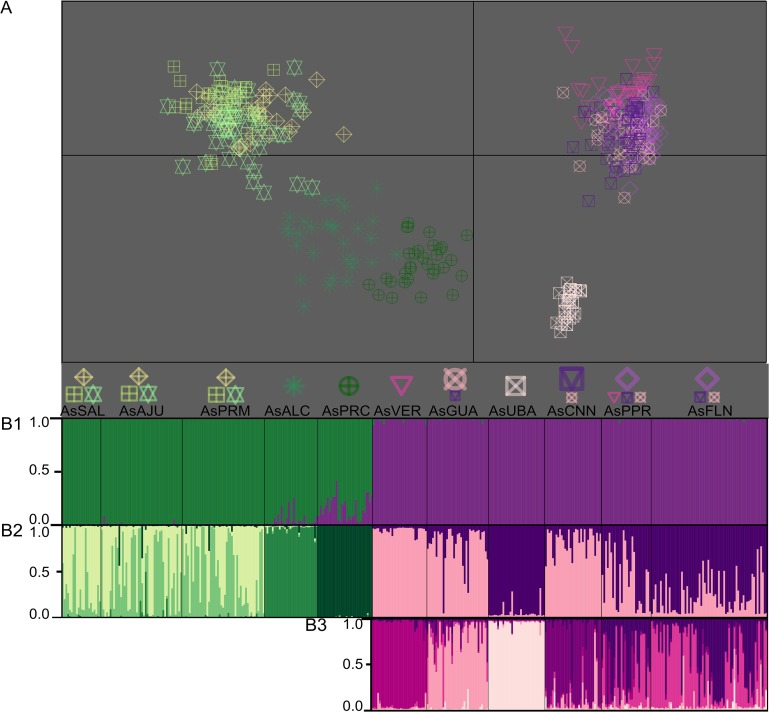
*A*. *schaueriana* population structure along the Brazilian coast. A) A scatterplot of the first two principal components of the multivariate analysis of DAPC [36], which resulted in a most likely k value of 10. Each symbol indicates the group to which each individual was assigned. The geographic origin of each individual is denoted for each locality sample such that more than 10% of the total number of individuals was composed of the inferred cluster. A larger symbol indicates that a cluster was predominant in the locality sample (pairwise cluster ratio larger than 1:5), whereas equal symbol sizes indicate similar cluster contributions to the total number of individuals in each sample. B1) Model-based clustering analyses [38,39] considering k = 2, where each individual is represented by a vertical line and each color refers to one inferred cluster; the posterior probability of group membership is indicated. These Bayesian analyses were extended to the inferred groups observed in B1, which correspond to samples north (green) and south (violet) of the NEESA. The posterior probabilities of group membership in this fine-scale analysis of AsN (k = 4) and AsS (k = 2) are shown in B2; further evaluations of AsS (k = 6) are displayed in B3.

A significant association was found between genetic and geographic distances, as indicated by the D and G_ST_ values for *A*. *germinans* and *A*. *schaueriana* (p < 0.01 for both species, with r_M_ = 0.777 and r_M_ = 0.696 for the former considering G_ST_ and D, and r_M_ = 0.755 and r_M_ = 0.830 for the latter). The observed IBD was even more evident when we evaluated the correlograms of r_M_ and classes of geographic distance (Fig. 3), which indicate a significant positive spatial structure. This IBD pattern was particularly evident when the multivariate DAPC analyses were considered. Using this method, we found that k was equal to 4 for *A*. *germinans*, with a substantial differentiation between TMD and the remaining samples (Fig. 4A). Also considering the DAPC analyses, for *A*. *schaueriana*, the most likely inferred k value was 10, and similarly, a clear pattern of divergence was observed between AsN and AsS (Fig. 5A).

**Fig 6:**
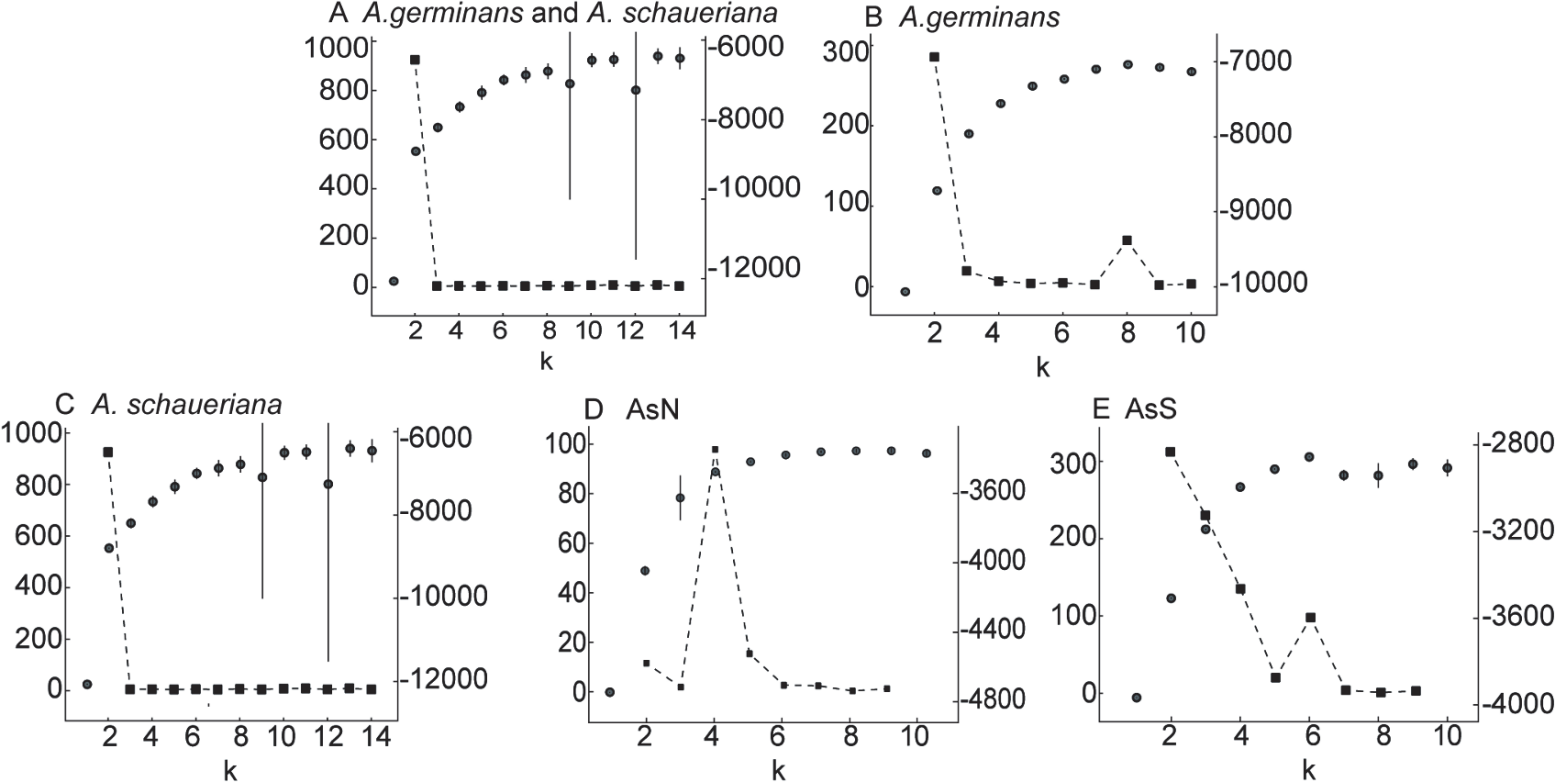
Bayesian inference of cluster number (k). The mean values of log posterior probability of data (lnL—isolated gray circles, left-axis [38]) and the ΔK *ad hoc* statistic (interconnected squares, right axis [40]) considering A) samples of both *A*. *germinans* and *A*. *schaueriana*, B) samples of *A*. *germinans*, C) individuals of *A*. *schaueriana* from all locales sampled, D) individuals of *A*. *schaueriana* from the northern cluster (AsN), and E) samples of *A*. *schaueriana* from the southern cluster (AsS).

Using the model-based method implemented in the Structure software, we observed two possible scenarios for *A*. *germinans*: k = 2 (AgN and AgS), as the most likely number of populations according to the ΔK approach, and k = 8, as an alternative scenario of finer genetic structure based on lnL and a smaller ΔK value peak (Fig. 6B). These results indicate the existence of a multiple-scale genetic structure. The fine-scale scenario suggests the divergence of samples across every evaluated sampling site, including AgPAa and AgPAb, which are separated by thousands of meters. For *A*. *schaueriana*, the most likely k value was 2 (AsN and AsS), based only on ΔK (Fig. 6C). Each of these groups was evaluated separately based on the pairwise G_ST_, D (Fig. 2), and DAPC (Fig. 5A) results. Finer-scale genetic structure was evident with AsN presenting k = 4 based on the ΔK values; with AsS presenting k = 2 based on the ΔK value; and with AsS showing k = 6 based on lnL and a smaller ΔK value peak (Fig. 6D and 6E). When inbreeding was considered, similar patterns of genetic structure were observed (S3 and S4 Figs.), despite the different inferred k values for both species (10 for *A*. *germinans* and 11 for *A*. *schaueriana*). Therefore, both species clearly exhibit variations in genetic structure on different geographic scales, which is in partial agreement with the IBD results as we found two inferred groups (AgPAa and AgPAb) that were clearly distinguished at the genetic level despite their geographic proximity. In all cases, a correspondence was observed between samples from each locality and the inferred clusters, which also allowed for the inference of admixed individuals and migrants (Figs. 4, 5, S1 and S2 Figs.).

The results of the hierarchical AMOVA indicated that the variation was primarily between AsN and AsS (45.88%). For this species, the among-group fixation indexes were significant, although they were lower in terms of the proportion of variation (Table 4) when fine-scale scenarios were considered (k = 4 for AsN and k = 5 or 8 for AsS). With respect to *A*. *germinans*, the variation between AgN and AgS, although substantial, was not supported by the permutation tests. However, when all seven samples were considered, a significant and substantial proportion of the variation could be explained by this level of the hierarchy (15.37%) (Table 4).

**Table 4:** Analysis of molecular variance (AMOVA) for *A*. *germinans* and *A*. *schaueriana* samples.

	A) As and Ag			B) AgN, AgS			C) AsN, AsS			D) Ag k = 4, As k = 5			E) Ag k = 4, As k = 8		
	%	F-FI		%	F-FI		%	F-FI		%	F-FI		%	F-FI	
**Among groups**	58.92	F_GT_	0.589	27.52	F_GT_	0.275^NS^	45.88	F_GT_	0.458	49.22	F_GT_	0.492	51.23	F_GT_	0.512
**Among populations within groups**	17.60	F_SG_	0.428	15.37	F_SG_	0.212	14.83	F_SG_	0.274	5.36	F_SG_	0.105	1.33	F_SG_	0.027
**Among individuals within populations**	3.54	F_IS_	0.150	7.07	F_IS_	0.123	9.85	F_IS_	0.250	11.38	F_IS_	0.250	11.89	F_IS_	0.250
**Within individuals**	19.94	F_IT_	0.800	50.04	F_IT_	0.499	29.44	F_IT_	0.705	34.04	F_IT_	0.659	35.55	F_IT_	0.644

Results of the hierarchical analysis of molecular variance (AMOVA) considering A) both *A*. *germinans* (Ag) and *A*. *schaueriana* (As) as groups, B) northern (AgN) and southern (AgS) to the northeast extreme of South America (NEESA) of *A*. *germinans* as groups, and C) samples of *A*. *schaueriana* north (AsN) and south (AsS) to the NEESA; samples of AgN with k = 4 and AsN with k = 2 (D) and k = 5 (E) according to Fig. 3. % indicates total variance; F-FI: fixation indexes considering infinite allele model (IAM). All results are significant (p < 0.005) except for those results presenting ^NS.^

### Ongoing hybridization between *A*. *germinans* and *A*. *schaueriana*

We observed that as expected, the observed differentiation between species was greater than the differentiation among samples within each species, with global G_ST_ and D values of 0.583 and 0.549, respectively, which were considerably higher than the values observed for individual species. Pairwise comparisons between samples, both within and between species, also indicated that the interspecific differences were much more pronounced than the intraspecific differences (Fig. 2). These results were also consistent with the hierarchical AMOVA approach, which revealed that the majority of the variation (58.92%) could be explained between species, although considerable and significant genetic variation remained between populations within each species (17.6%) (Table 4).

**Fig 7:**
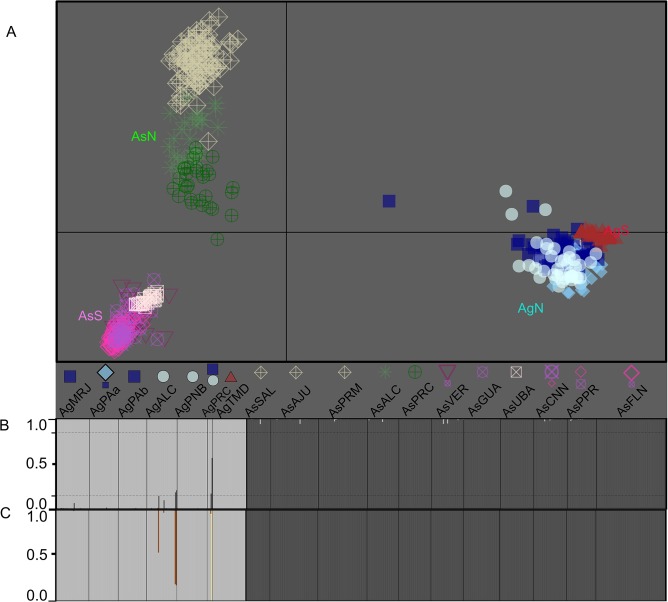
Analyses of ongoing hybridization between *A*. *germinans* and *A*. *schaueriana*. A) A scatterplot of the first two principal components of the multivariate analysis of DAPC [36]. Each symbol indicates the group to which each individual was assigned. The geographic origin of each individual is denoted for each locality sample such that more than 10% of the total number of individuals is composed of the inferred cluster. A larger symbol indicates that a cluster was predominant in the locality sample (pairwise cluster ratio larger than 1:5), whereas equal symbol sizes indicate similar cluster contributions to the total individuals of each sample. B) Model-based clustering analyses [38,39] considering k = 2, where each individual is represented by a vertical line and each color refers to one cluster; the posterior probability of group membership is indicated. The dashed horizontal lines denote the arbitrary threshold of 0.15, which was used as a sign of possible interspecific hybridization. C) Posterior probability of the model-based approach for identifying species hybrids [45]. Each vertical line also refers to an individual, and different colors indicate distinct classes of individuals: light gray: “pure” *A*. *germinans*; dark gray: “pure” *A*. *schaueriana*; dark orange: *A*. *germinans* second-generation backcrossed individuals; and yellow: F1 interspecific hybrid.

Regarding the DAPC results, we observed an optimum of 11 clusters, revealing the differences between *A*. *germinans* and *A*. *schaueriana* (Fig. 7A). The patterns of genetic structure within each species were consistent with the multi-scale results described above when each taxon was evaluated separately, including a clear divergence between the northern and southern samples for both *A*. *germinans* and *A*. *schaueriana*. Using DAPC, one F_1_ interspecific hybrid from AgPRC (Fig. 7A), which was situated between the two primary clusters, was evident despite the individual assignment. Based on two different model-based approaches, we uncovered more evidence that these species are hybridizing and that these hybrids are fertile. Considering the model implemented in the Structure software [38], the most likely k value was 2 based only ΔK (Fig. 6). The differentiation between *A*. *germinans* and *A*. *schaueriana* remained obvious; however, compared with the DAPC results, there was more evidence supporting ongoing hybridization between these species. Considering the arbitrary threshold of the assignment probability of 0.15, we identified four individuals that were most likely the result of matings between *A*. *germinans* and *A*. *schaueriana*. When placed between the two primary clusters in the multivariate analysis (Fig. 7A), the same interspecific hybrid individual presented assignment probabilities of 0.566 and 0.434 for each inferred group, which is a reliable indication of an F_1_ hybrid (Fig. 7B). Considering each species as a “pure category,” we used another model-based approach to verify the presence of different classes of up to two generation hybrids. As the use of Jeffrey-like and uniform priors yielded slightly different results, with the latter providing more conservative outcomes for the numbers and likelihood of hybrids, we considered only the uniform distribution as prior. The individual inferred as a likely hybrid from PRC considering the Structure and DAPC analyses was unequivocally assigned as an F_1_ hybrid with a posterior probability of 1.0. We also observed that two plants from AgALC were likely descendants of a cross between an F_1_ individual and an *A*. *germinans* genitor (unidirectional backcross—probability > 0.80) (Fig. 7C). No sign of hybridization was evident when considering individuals identified as *A*. *schaueriana*, indicating a unidirectional introgression process (Fig. 7 and S5 Fig.).

## Discussion

### *A*. *schaueriana* microsatellite markers

We characterized the first set of microsatellites for *A*. *schaueriana* to complement the molecular markers developed for *A*. *germinans* [22–24]. We observed a variable degree of polymorphism among loci and a high transferability rate between these species (Table 2). The present study demonstrates that these markers are valuable molecular tools that can be used to address a wide range of questions regarding these species.

### Mating system and intraspecific genetic diversity and structure in *A*. *germinans* and *A*. *schaueriana*

Considering the progeny array of *A*. *schaueriana*, we found an intermediate outcrossing rate, which significantly departed from both 0 and 1, indicating that this population has a mixed mating system [48]. Comparing the t_a_ values estimated for *A*. *schaueriana* from AsAJU (0.529), we observed that this variable closely approximated the *t*_m_ estimated via the progeny array (0.542 ± 0.062), indicating that inbreeding is a consistent feature of this population across generations. These results are consistent with the reproductive biology of these species as this tree is self-compatible with a pollinator-dependent generalist pollination system [17], similar to the gray mangrove *A*. *marina* [49], which is widespread throughout the IWP region. Considering the three *A*. *germinans* progeny arrays from the Pacific coast, we found comparable t_m_ values (0.583 ± 0.09, 0.774 ± 0.09 and 0.770 ± 0.12 for each array) and proportions of progeny that shared the same male parent for two of the three progeny, indicating that this species may also exhibit moderate levels of self-fertilization. Considering the evaluations of the *A*. *germinans* mating system, substantially lower biparental inbreeding was apparent [50]. Unfortunately, these previous analyses are not easily comparable with ours as we used more loci (13) than Nettel et al. [50] (six) and the difference between t_m_ and t_s_ is sensitive to the number of loci, with more markers resulting in values closer to the true difference between these parameters [47]. However, using a different method, Cerón-Souza and colleagues [10] suggested that biparental inbreeding may indeed be relevant to the *A*. *germinans* mating system, indicating that inbreeding in these species could be affected by both selfing and mating among relatives. Taken together, this evidence suggests that *A*. *germinans*, whose flowers are visited by a wide range of insects [18], and *A*. *schaueriana* generally exhibit similar mixed mating systems.

For both species throughout the sampling range, we also found evidence of a mixed mating system (average t_a_ of 0.704 for *A*. *germinans* and 0.610 for *A*. *schaueriana*) and varying degrees of inbreeding for most of the evaluated samples (Table 3). Previous studies of *A*. *germinans* [10,15], *A*. *marina* [51–53] and *A*. *bicolor* [15] are consistent with these findings. However, we did not observe evidence for large amounts of inbreeding in the southernmost samples of *A*. *germinans* or in the southernmost or northernmost samples of *A*. *schaueriana*, similar to the findings reported for *A*. *marina* [51], which may be due to ecological, geographical and/or historical differences between these species. This mixed mating system involving a broad range of pollinators may represent an adaptation [54] that may be particularly important to mangrove trees as colonizers [1,55] in terms of reproductive assurance [48]. These findings suggest that self-fertilization—and likely mating between relatives—is a frequent feature throughout the genus, and has had substantial influence on the genetic structure of these species due to pollen dispersal restrictions and, consequently, limited gene flow.

The genetic structure patterns we observed for both species support these reproduction-related findings. We argue that this mixed mating system showing biparental inbreeding influences the substantial genetic variation observed among the samples, despite the different global measures of population differentiation. In particular, D was considerably lower for *A*. *schaueriana*, which could be explained by its low diversity within populations, likely due to high levels of inbreeding and/or mating between relatives [31]. These summary values are comparable to those previously reported for *A*. *germinans* [10,15], *A*. *bicolor* [15] and *A*. *marina* [52]. Pairwise comparisons between the different population differentiation measurements were significantly correlated for every pair of samples (Fig. 2). This finding indicates that significant genetic differences exist even for geographically close samples despite low values for G_ST_ and D, which is consistent with multivariate and model-based assignment analyses, which showed very similar results.

Considering the fine-scale genetic structures from the DAPC and Bayesian population assignment methods, with k = 8 for *A*. *germinans*, k = 4 for AsN, and k = 6 for AsS, we observed well-defined groups at both the local and regional scales. This was true even where there was no clear physical barrier to pollen and propagule dispersal, for instance, among AsSAL, AsAJU, AsPRM and AsALC or among AgPAa, AgPAb or AgALC, which are samples collected within the world’s largest continuous area of mangrove forests [56] (Figs. 4, 5, S1 and S2 Figs.). AMOVA results also supported this fine-scale genetic structure pattern, as both species had significant fixation index values when the variation among populations within groups was considered (Table 4). For instance, the clear differentiation between AgPAa and AgPAb was striking. These populations are geographically close, within a few kilometers of one another, but are subject to distinct hydrographic regimes [19]. In this case, assuming microsatellites as neutral genetic markers, we argue that tide plays an important role in shaping the genetic diversity of *Avicennia*. Despite the putative neutrality of these microsatellites, this phenomenon may be enhanced by selective pressures acting on these neutral markers via the hitchhiking effect [57], leading to even more differentiated populations. Thus, hydrographic patterns with a low-frequency tide play an important role as barriers to dispersal, as expected based on the water-based dispersal of mangrove propagules. Only one likely admixed individual was identified within the AgPAa samples, indicating that a limited pollen dispersal constraint also exists, which may be enhanced by the mating systems of these species. In this sense, despite the evidence of LDD for *A*. *germinans* [20], our results indicate that regardless of their significant evolutionary consequences [58,59], these phenomena are sufficiently rare that *A*. *germinans* and *A*. *schaueriana* populations present divergent gene pools even at the local and regional geographic scales.

Therefore, both the present and previous analyses indicates that the pollen and propagule dispersal constraints of the mating system of these species influence their genetic structure at small geographic scales. However, despite the genetic divergence at local and regional spatial scales, a positive relation exists between genetic and geographic distances, leading to an IBD pattern (Fig. 3). Similar to that reported for *R*. *mangle* [8,9] and *Hibiscus pernambucensis* [60], substantial divergence exists between samples from locales north and south of the NEESA for both *A*. *germinans* and *A*. *schaueriana*. According to the majority of the approaches we used, the most pronounced indications of genetic structure emerged when this divergence was considered. Pairwise G_ST_ and D values were the highest for pairs of samples between these regions (Fig. 2). The DAPC analyses suggest that the greatest differentiation was found between these northern and southern groups, compared with samples within each cluster (Figs. 4A and 5A). The ΔK values were also the highest for k = 2 for both *A*. *germinans* and *A*. *schaueriana* (Fig. 6). For *A*. *schaueriana*, the amount of variation between these groups was 45.88% according to AMOVA, and lower variation, although significant, was added when we considered more levels of the hierarchy. However, for *A*. *germinans*, the between-group fixation indexes were not significant (Table 4), regardless of the mutation model assumed, most likely because AgTMD was the only sample of AgS and because this species exhibits substantial local and regional genetic structure.

As has been previously discussed [8,9,60], the bifurcation of the southern branch of the South Equatorial Current (SEC) into the Brazil Current (BC) and the cross-equatorial North Brazil Current (NBC—northwestward) (Fig. 1–[61]) constrains the movement of propagules between the northern and southern groups. In addition to acting as a barrier, the branching of the SEC, as well as the high velocity of the NBC compared with the BC [61], favor the migration of individuals from the southern to the northern regions, leading to a higher frequency of admixture events in the north. This pattern is readily observed in the Structure software results (Figs. 4, 5, S1 and S2 Figs.). For instance, considering *A*. *germinans*, for k = 2, the absence of admixed individuals in the TMD sample and their presence in the AgPRC samples follow the direction of the NBC marine current. *A*. *schaueriana* exhibits the same pattern: for k = 2, there is little evidence of admixture in the southern group, whereas substantial evidence of admixed individuals exists in AsALC and AsPRC samples. Moreover, regarding AsN, there are indications that propagule flow follows the direction of the NBC. In contrast, AsS shows a more complex pattern in which no single direction of admixed individuals is apparent for k = 2 or 6 (Fig. 5B). This result may be related to the slower mean velocity of the BC and its seasonal variations in velocity and direction [61] in southern and southeastern Brazil, where AsGPM, AsUBA and AsCNN were sampled. Furthermore, not only the direction but also the speed and seasonal variance of the marine currents play an important role in the genetic diversity of these sea-dispersed plants at multiple geographic scales. However, the role of marine currents in shaping mangrove genetic diversity is not restricted to the Atlantic coast of South America. Along the northwestern coast of Mexico, for instance, the California Current and the El Niño Southern Oscillation could explain the patterns of gene flow observed for *R*. *mangle* [62]. Regarding the IWP biogeographic region, links between marine currents and genetic variation have been reported for several different mangrove taxa, including *R*. *mucronata* Lam. (Rhizophoraceae) [63], *Ceriops tagal* (Perr.) (Rhizophoraceae) [64] *Kandelia candel* (L.) Druce (Rhizophoraceae) [65] and *Lumnitzera racemosa* Willd (Combretaceae) [66]. Considered together with our findings, this body of evidence suggests that the influence of surface marine currents on genetic variation in mangroves, and likely other sea-dispersed organisms, is a general feature of evolution.

### Ongoing hybridization between *A*. *germinans* and *A*. *schaueriana*

There is evidence of an ancient introgression between *A*. *germinans* and *A*. *bicolor*, a species with a limited distribution along the Pacific coast of Central America [1], as well as chloroplast capture between *A*. *germinans* and *A*. *schaueriana* in the Atlantic basin [15]. Although we were unable to evaluate any chloroplast genome markers, we found evidence that ongoing hybridization is indeed occurring between these species within the *A*. *germinans* and *A*. *schaueriana* sympatry zone. To the best of our knowledge, this is the first report of ongoing hybridization in *Avicennia*.

These mangrove species present distinct gene pools, which is evident when these species are compared using the pairwise G_ST_ and D values between samples or using the DAPC approach, in which the genetic diversity between each species is much greater than the variation within each species (Figs. 2 and 7). Moreover this is consistent with the AMOVA at the interspecific level of the hierarchy (Table 4). In addition to differentiating these two species, these analyses also recapitulate the previously discussed genetic structure within each species. The multivariate assignment approach suggests a likely interspecific hybrid identified as *A*. *germinans* from AgPRC, which is graphically located between the clusters of individuals representing each species (Fig. 7A). Further Bayesian evaluations not only corroborated the likely hybrid identified using DAPC as an F_1_ hybrid but also assigned other *A*. *germinans* individuals from AgALC as likely second-generation backcrosses between F_1_ hybrids and this species (Fig. 7C); these results also indicate that eventual hybrids are fertile, at least when mating with *A*. *germinans*. The Bayesian methods also revealed that this introgression is likely unidirectional as only individuals identified as *A*. *germinans* showed evidence of ongoing admixture between the species (as illustrated in S5 Fig.). These species share both pollinators [17,18] and flower traits [1,17], and a reproductive phenological overlap has been reported [19], indicating that hybridization is possible between these species. Additionally, during our sampling, *A*. *germinans* was more commonly found in Alcântara, Maranhão, Brazil [19], but was much less abundant in Paracuru, Ceará, throughout our sampling. These findings indicate that post-zygotic mechanisms are related to this asymmetric hybridization, as was previously observed for other mangrove genera [12,67]. We consider the mechanisms that generate and maintain this unidirectional introgression worthy of further investigation.

The ancient introgression between *A*. *bicolor* and *A*. *germinans* observed along the Pacific coast of Central America [15] was related to the higher diversity in that region than the Atlantic coast [10,15]. However, when considering the South American Atlantic coast, this association is not possible as AgAJU and AgPRC do not show higher genetic diversities in terms of the number of alleles or expected heterozygosity than locales with no evidence of hybridization (Table 3). Therefore, we argue that ongoing interspecific hybridization did not increase the genetic diversity of *A*. *germinans* in the samples we evaluated. This could indicate that the observed interspecific mating was recent and that too little time has passed for *A*. *schaueriana* alleles to spread among the *A*. *germinans* populations or, alternatively, that natural selection is acting against the hybrid, reducing the spread of its alleles. Again, although we may hypothesize about the consequences of the observed hybridization, we encourage further investigations into the effects of interspecific hybridization on not only the genetic diversity of populations but also individual phenotype and fitness.

### Concluding remarks

Using the first set of microsatellites developed for *A*. *schaueriana* as well as markers previously developed for *A*. *germinans*, we studied three biological aspects of these species: the existence of hybridization between these species, their mating systems, and the organization of neutral genetic variants at different geographic scales. Our results suggest that an interplay between intrinsic (e.g., mating system, limited pollen and propagule dispersal but not hybridization) and extrinsic factors (e.g., marine currents and tide) shape the genetic diversity of *A*. *germinans* and *A*. *schaueriana*, leading to genetic diversity structured at the micro-, meso- and macro-scales for both of these *Avicennia* species.

Similar patterns of neutral genetic variation organization have been observed in different taxa of mangrove species, which is likely related to their shared colonization ability [1,55]. For instance, a large-scale genetic structure was reported for *Rhizophora mangle* [8,9]; however, its genetic diversity is also locally and regionally organized on smaller scales [10]. This multiple-scale genetic structure is observed in these phylogenetically distant species regardless of the different propagule features of each genus [5,6]. The generality or specificity of these findings and the mechanisms that generate these similar patterns remains to be evaluated in other mangrove species, perhaps using a landscape genetics approach [68]. However, this investigation demonstrates a consistent, multiple-geographic-scale genetic structure pattern for two Neotropical *Avicennia* species. Moreover, we are aware that these different spatial scales imply distinct temporal scales of evolutionary response. Considering this, future efforts will be focused on elucidating the processes that generate and maintain these patterns.

## Supporting Information

S1 Table Pairwise G_ST_ of samples genetic differentiation comparing the effect of null alleles for *A*. *germinans*. (DOCX)

S2 Table Pairwise G_ST_ of samples genetic differentiation comparing the effect of null alleles for *A*. *schaueriana*. (DOCX)

S3 Table Pairwise measurements of the genetic variation among samples in terms of G_ST_ (Nei 1973), D (Jost 2008) regarding A) *A*. *germinans* and *A*. *schaueriana;* B) *A*. *germinans* and C) *A*. *schaueriana*. (DOCX)

S1 File Datasets used in this study. (XLSX)

S1 Fig Representation of *Avicennia germinans* multiple-geographic-scale genetic structure using STRUCTURE, considering multiple-geographic-scale genetic structure. Photographs of two geographically close environments under different tide regimes (photos by Gustavo Maruyama Mori).
(PNG)

S2 Fig Representation of *Avicennia schaueriana* multiple-geographic-scale genetic structure using STRUCTURE considering multiple-geographic-scale genetic structure. (PNG)

S3 Fig Bayesian clustering analysis of *A*. *germinans* populations sampled along the Brazilian coast considering inbreeding using the method implemented in InStruct [42]. (PNG)

S4 Fig Bayesian clustering analysis of *A*. *schaueriana* populations sampled along the Brazilian coast considering inbreeding using the method implemented in InStruct [42]. (PNG)

S5 Fig Representation of unidirectional gene flow between *Avicennia schaueriana* and *A*. *germinans* indicating asymmetric hybridization (photos by Gustavo Maruyama Mori). (PNG)
