# Supplementary material for: Multiple-Geographic-Scale Genetic Structure of Two Mangrove Tree Species: The Roles of Mating System, Hybridization, Limited Dispersal and Extrinsic Factors
Source: PLoS One. 2015 Feb 27;10(2):e0118710. doi: 10.1371/journal.pone.0118710 (PMC4344226; doi:10.1371/journal.pone.0118710)
Supplement: S1 Table — (DOCX) [file pone.0118710.s001.docx]

**Table S1.** Pairwise G_ST_ of samples genetic differentiation comparing the effect of null alleles for *A. germinans.*

|  | AgALC | AgMRJ | AgPAa | AgPAb | AgTMD | AgPNB | AgPRC |
| --- | --- | --- | --- | --- | --- | --- | --- |
| AgALC |  | 0.15248 | 0.194459 | 0.105602 | 0.52152 | 0.08938 | 0.116662 |
| AgMRJ | 0.154614 |  | 0.197007 | 0.056699 | 0.422091 | 0.263621 | 0.166099 |
| AgPAa | 0.19817 | 0.199035 |  | 0.16896 | 0.58455 | 0.330845 | 0.234802 |
| AgPAb | 0.111851 | 0.055144 | 0.174333 |  | 0.422717 | 0.19299 | 0.128432 |
| AgTMD | 0.544967 | 0.44249 | 0.601351 | 0.448288 |  | 0.626313 | 0.606566 |
| AgPNB | 0.090494 | 0.267037 | 0.339511 | 0.200083 | 0.649346 |  | 0.247768 |
| AgPRC | 0.131166 | 0.194667 | 0.248797 | 0.149596 | 0.639252 | 0.261579 |  |

Pairwise G_ST_ regarding *A. germinans* samples considering (above diagonal) and not considering null alleles (below diagonal) using the method implemented in FreeNA [27].
