# Supplementary material for: Multiple-Geographic-Scale Genetic Structure of Two Mangrove Tree Species: The Roles of Mating System, Hybridization, Limited Dispersal and Extrinsic Factors
Source: PLoS One. 2015 Feb 27;10(2):e0118710. doi: 10.1371/journal.pone.0118710 (PMC4344226; doi:10.1371/journal.pone.0118710)
Supplement: S2 Table — (DOCX) [file pone.0118710.s002.docx]

**Table S2.** Pairwise G_ST_ of samples genetic differentiation comparing the effect of null alleles for *A. schaueriana.*

|  | AsAJU | AsPRM | AsSAL | AsGPM | AsVER | AsALC | AsPPR | AsFLN | AsCNN | AsUBA | AsPRC |
| --- | --- | --- | --- | --- | --- | --- | --- | --- | --- | --- | --- |
| AsAJU |  | 0.013171 | 0.016007 | 0.511992 | 0.522756 | 0.283788 | 0.513524 | 0.561715 | 0.519784 | 0.595578 | 0.436597 |
| AsPRM | 0.006418 |  | 0.01708 | 0.517269 | 0.526272 | 0.310654 | 0.515317 | 0.561235 | 0.523999 | 0.597374 | 0.449071 |
| AsSAL | 0.006666 | 0.007095 |  | 0.553491 | 0.567828 | 0.304543 | 0.55792 | 0.602303 | 0.563873 | 0.665052 | 0.510289 |
| AsGPM | 0.531818 | 0.531387 | 0.55842 |  | 0.266205 | 0.465435 | 0.147969 | 0.174467 | 0.159345 | 0.451664 | 0.556271 |
| AsVER | 0.541768 | 0.539382 | 0.573117 | 0.293845 |  | 0.457831 | 0.242601 | 0.318235 | 0.279517 | 0.569099 | 0.609898 |
| AsALC | 0.30497 | 0.323484 | 0.309449 | 0.48163 | 0.477107 |  | 0.47137 | 0.513226 | 0.480749 | 0.570962 | 0.500903 |
| AsPPR | 0.529528 | 0.523881 | 0.558169 | 0.169841 | 0.264906 | 0.482366 |  | 0.070697 | 0.179505 | 0.428488 | 0.579456 |
| AsFLN | 0.585392 | 0.579374 | 0.615636 | 0.206552 | 0.35121 | 0.537977 | 0.075175 |  | 0.203604 | 0.36852 | 0.595592 |
| AsCNN | 0.53547 | 0.53481 | 0.567241 | 0.172109 | 0.29008 | 0.495385 | 0.198356 | 0.228261 |  | 0.437516 | 0.588892 |
| AsUBA | 0.612865 | 0.60732 | 0.67098 | 0.478236 | 0.598174 | 0.587784 | 0.454288 | 0.416079 | 0.484246 |  | 0.68663 |
| AsPRC | 0.456768 | 0.459293 | 0.51787 | 0.575367 | 0.629219 | 0.518806 | 0.596456 | 0.625324 | 0.613709 | 0.712541 |  |

Pairwise G_ST_ regarding *A. schaueriana* samples considering (above diagonal) and not considering null alleles (below diagonal) using the method implemented in FreeNA [27].
