# Supplementary material for: Multiple-Geographic-Scale Genetic Structure of Two Mangrove Tree Species: The Roles of Mating System, Hybridization, Limited Dispersal and Extrinsic Factors
Source: PLoS One. 2015 Feb 27;10(2):e0118710. doi: 10.1371/journal.pone.0118710 (PMC4344226; doi:10.1371/journal.pone.0118710)
Supplement: S3 Table — (DOCX) [file pone.0118710.s003.docx]

**Table S3.** Pairwise measurements of the genetic variation among samples in terms of G_ST_ (Nei 1973), D (Jost 2008) regarding A) *A. germinans* and *A. schaueriana;* B) *A. germinans* and C) *A. schaueriana.*

1. ***A. germinans* and *A. schaueriana***

| GST (above diagona), D (below diagonal) | | | | | | | | | | | | | | | | | | |
| --- | --- | --- | --- | --- | --- | --- | --- | --- | --- | --- | --- | --- | --- | --- | --- | --- | --- | --- |
|  | AgMRJ | AgPAa | AgPAb | AgALC | AgPNB | AgPRC | AgTMD | AsSAL | AsAJU | AsPRM | AsALC | AsPRC | AsVER | AsGUA | AsUBA | AsCNN | AsPPR | AsFLN |
| AgMRJ |  | 0.0923 | 0.03 | 0.0952 | 0.1646 | 0.1118 | 0.2528 | 0.4078 | 0.4008 | 0.3959 | 0.3979 | 0.4629 | 0.4482 | 0.4481 | 0.499 | 0.4447 | 0.4466 | 0.4583 |
| AgPAa | 0.1353 |  | 0.0697 | 0.079 | 0.1563 | 0.1234 | 0.3741 | 0.4677 | 0.4605 | 0.455 | 0.4614 | 0.5236 | 0.51 | 0.5077 | 0.5627 | 0.5061 | 0.5078 | 0.519 |
| AgPAb | 0.0601 | 0.1116 |  | 0.0646 | 0.1129 | 0.0824 | 0.2589 | 0.414 | 0.407 | 0.402 | 0.4077 | 0.4708 | 0.4576 | 0.4562 | 0.5075 | 0.4568 | 0.456 | 0.4669 |
| AgALC | 0.1923 | 0.0961 | 0.1222 |  | 0.0481 | 0.062 | 0.3597 | 0.436 | 0.4296 | 0.4239 | 0.4237 | 0.4891 | 0.4794 | 0.478 | 0.533 | 0.4822 | 0.4796 | 0.4902 |
| AgPNB | 0.2517 | 0.1713 | 0.1487 | 0.0285 |  | 0.0958 | 0.4387 | 0.5334 | 0.5251 | 0.5188 | 0.5228 | 0.5861 | 0.5729 | 0.57 | 0.6321 | 0.5745 | 0.572 | 0.5832 |
| AgPRC | 0.2123 | 0.1774 | 0.1403 | 0.0856 | 0.0909 |  | 0.3231 | 0.3913 | 0.3813 | 0.3797 | 0.3628 | 0.4299 | 0.4271 | 0.4225 | 0.4826 | 0.4284 | 0.4283 | 0.4354 |
| AgTMD | 0.22 | 0.3176 | 0.2364 | 0.3743 | 0.2898 | 0.2826 |  | 0.6823 | 0.6685 | 0.6648 | 0.67 | 0.7633 | 0.745 | 0.7386 | 0.8137 | 0.7352 | 0.7407 | 0.7541 |
| AsSAL | 0.9324 | 0.9723 | 0.9308 | 0.9053 | 0.9456 | 0.7277 | 0.8949 |  | 0.0045 | 0.0074 | 0.1918 | 0.3351 | 0.407 | 0.385 | 0.4838 | 0.3919 | 0.387 | 0.4121 |
| AsAJU | 0.9356 | 0.9769 | 0.9357 | 0.9117 | 0.9533 | 0.7122 | 0.8825 | 0.0003 |  | 0.0054 | 0.1809 | 0.3059 | 0.3883 | 0.3678 | 0.4604 | 0.3702 | 0.3695 | 0.3922 |
| AsPRM | 0.9334 | 0.9753 | 0.9315 | 0.9075 | 0.9489 | 0.7203 | 0.9044 | 0.0014 | 0.0017 |  | 0.2025 | 0.308 | 0.3935 | 0.3746 | 0.4615 | 0.3776 | 0.3721 | 0.3919 |
| AsALC | 0.8922 | 0.9681 | 0.9194 | 0.8399 | 0.9034 | 0.6012 | 0.8877 | 0.0656 | 0.0612 | 0.0852 |  | 0.3206 | 0.3278 | 0.3247 | 0.4201 | 0.3359 | 0.3285 | 0.3465 |
| AsPRC | 0.9348 | 0.9369 | 0.9314 | 0.8483 | 0.8235 | 0.6516 | 0.8975 | 0.0996 | 0.0884 | 0.1016 | 0.1082 |  | 0.4197 | 0.3466 | 0.4826 | 0.385 | 0.3663 | 0.3879 |
| AsVER | 0.9119 | 0.9244 | 0.9178 | 0.8474 | 0.8111 | 0.6726 | 0.8866 | 0.2002 | 0.1932 | 0.2102 | 0.1345 | 0.1123 |  | 0.1914 | 0.4328 | 0.1815 | 0.1644 | 0.221 |
| AsGUA | 0.9196 | 0.9318 | 0.925 | 0.8602 | 0.8244 | 0.6613 | 0.8679 | 0.1804 | 0.179 | 0.1968 | 0.1388 | 0.0689 | 0.0291 |  | 0.3244 | 0.1001 | 0.102 | 0.1223 |
| AsUBA | 0.9348 | 0.9429 | 0.9305 | 0.8814 | 0.8476 | 0.7029 | 0.8813 | 0.1873 | 0.1721 | 0.1858 | 0.1438 | 0.0843 | 0.0831 | 0.0457 |  | 0.3228 | 0.2917 | 0.2749 |
| AsCNN | 0.8975 | 0.9243 | 0.9396 | 0.9124 | 0.8868 | 0.7014 | 0.8167 | 0.1805 | 0.167 | 0.1864 | 0.1551 | 0.1018 | 0.0311 | 0.0221 | 0.0494 |  | 0.1179 | 0.1312 |
| AsPPR | 0.9204 | 0.9335 | 0.9222 | 0.8771 | 0.8458 | 0.693 | 0.8823 | 0.1678 | 0.1649 | 0.1752 | 0.1423 | 0.0813 | 0.0359 | 0.013 | 0.0301 | 0.021 |  | 0.0389 |
| AsFLN | 0.9263 | 0.9377 | 0.9235 | 0.874 | 0.8406 | 0.6834 | 0.8891 | 0.188 | 0.1814 | 0.1831 | 0.1422 | 0.0866 | 0.0386 | 0.0173 | 0.0215 | 0.0214 | 0.0031 |  |

1. ***A. germinans***

| D | | | | | | | |
| --- | --- | --- | --- | --- | --- | --- | --- |
|  | AgMRJ | AgPAa | AgPAb | AgALC | AgPNB | AgPRC | AgTMD |
| AgMRJ |  | 0.111 | 0.0279 | 0.0855 | 0.1556 | 0.1124 | 0.283 |
| AgPAa | 0.1491 |  | 0.0949 | 0.1111 | 0.2029 | 0.1292 | 0.4216 |
| AgPAb | 0.0494 | 0.1252 |  | 0.0606 | 0.1103 | 0.0856 | 0.2812 |
| AgALC | 0.1563 | 0.137 | 0.1047 |  | 0.0483 | 0.0709 | 0.3637 |
| AgPNB | 0.2163 | 0.216 | 0.1353 | 0.0277 |  | 0.1266 | 0.4691 |
| AgPRC | 0.221 | 0.1872 | 0.1516 | 0.1094 | 0.1406 |  | 0.3012 |
| AgTMD | 0.268 | 0.3743 | 0.2575 | 0.4091 | 0.3425 | 0.2711 |  |

1. ***A. schaueriana***

| D | | | | | | | | | | | |
| --- | --- | --- | --- | --- | --- | --- | --- | --- | --- | --- | --- |
|  | AsSAL | AsAJU | AsPRM | AsALC | AsPRC | AsVER | AsGUA | AsUBA | AsCNN | AsPPR | AsFLN |
| AsSAL |  | 0.0058 | 0.0067 | 0.1842 | 0.3313 | 0.3924 | 0.377 | 0.475 | 0.3843 | 0.3797 | 0.4075 |
| AsAJU | 0.0005 |  | 0.0049 | 0.1827 | 0.2991 | 0.3835 | 0.3706 | 0.4631 | 0.3738 | 0.3734 | 0.3989 |
| AsPRM | 0.0013 | 0.0016 |  | 0.1989 | 0.3036 | 0.3846 | 0.3726 | 0.4593 | 0.3759 | 0.3711 | 0.394 |
| AsALC | 0.0652 | 0.068 | 0.0882 |  | 0.3413 | 0.3145 | 0.3151 | 0.4104 | 0.3262 | 0.3196 | 0.3399 |
| AsPRC | 0.1036 | 0.0855 | 0.1012 | 0.1251 |  | 0.4469 | 0.3937 | 0.5303 | 0.428 | 0.413 | 0.4401 |
| AsVER | 0.1958 | 0.2006 | 0.2116 | 0.1224 | 0.1316 |  | 0.1843 | 0.4236 | 0.1755 | 0.1588 | 0.2163 |
| AsGUA | 0.1807 | 0.1901 | 0.2025 | 0.1277 | 0.0875 | 0.0265 |  | 0.32 | 0.0969 | 0.0991 | 0.1211 |
| AsUBA | 0.1895 | 0.1848 | 0.1939 | 0.1334 | 0.1045 | 0.0761 | 0.0416 |  | 0.3178 | 0.288 | 0.2746 |
| AsCNN | 0.1823 | 0.1798 | 0.1941 | 0.1433 | 0.1229 | 0.0285 | 0.0201 | 0.045 |  | 0.1148 | 0.1292 |
| AsPPR | 0.1701 | 0.1776 | 0.1832 | 0.1315 | 0.1013 | 0.0329 | 0.0118 | 0.0274 | 0.0191 |  | 0.0391 |
| AsFLN | 0.1903 | 0.1942 | 0.1914 | 0.132 | 0.1068 | 0.0355 | 0.0157 | 0.0195 | 0.0196 | 0.0029 |  |
