# Supplementary figures and images for: Multiple-Geographic-Scale Genetic Structure of Two Mangrove Tree Species: The Roles of Mating System, Hybridization, Limited Dispersal and Extrinsic Factors
Source: PLoS One. 2015 Feb 27;10(2):e0118710. doi: 10.1371/journal.pone.0118710 (PMC4344226; doi:10.1371/journal.pone.0118710)

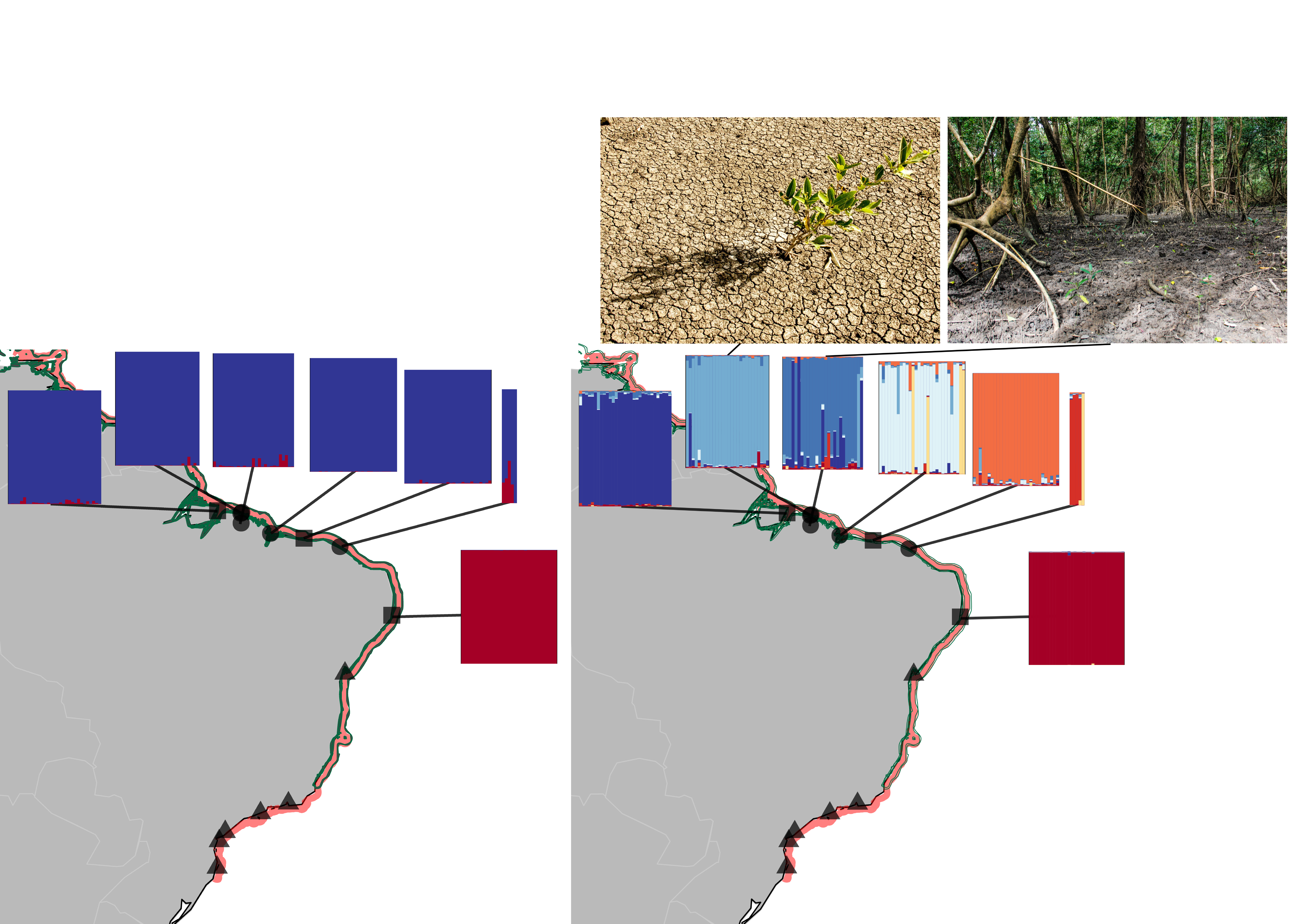

Supplement: S1 Fig — Photographs of two geographically close environments under different tide regimes (photos by Gustavo Maruyama Mori). (PNG) [file pone.0118710.s005.png]

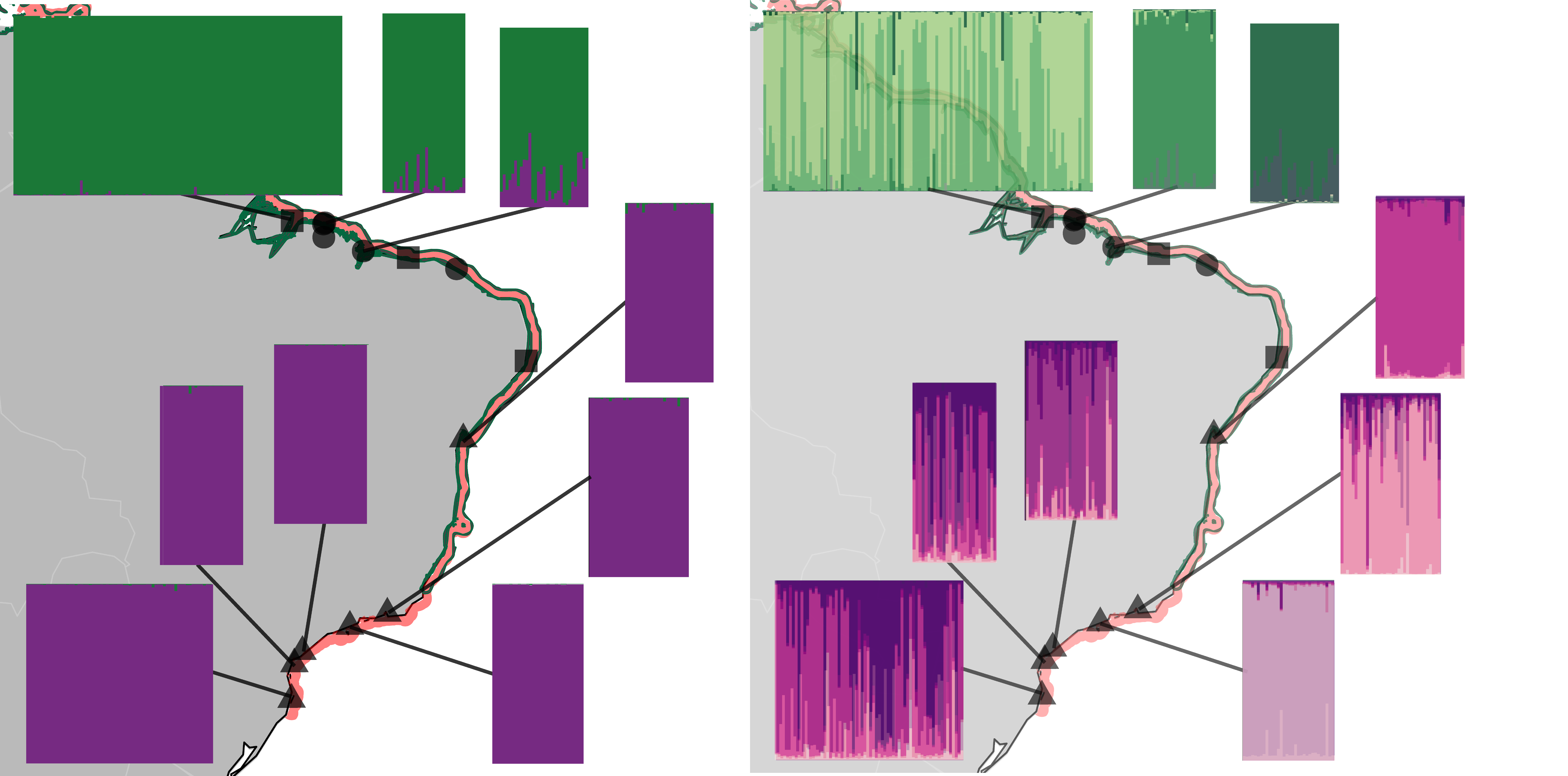

Supplement: S2 Fig — (PNG) [file pone.0118710.s006.png]

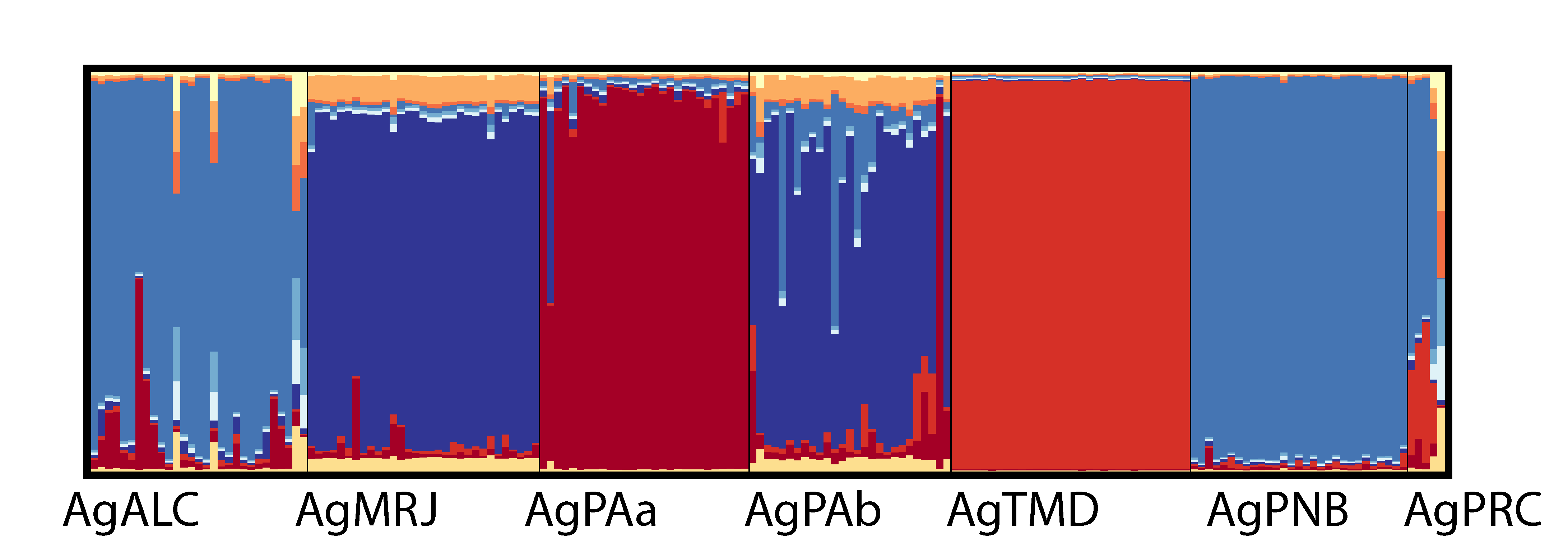

Supplement: S3 Fig — (PNG) [file pone.0118710.s007.png]

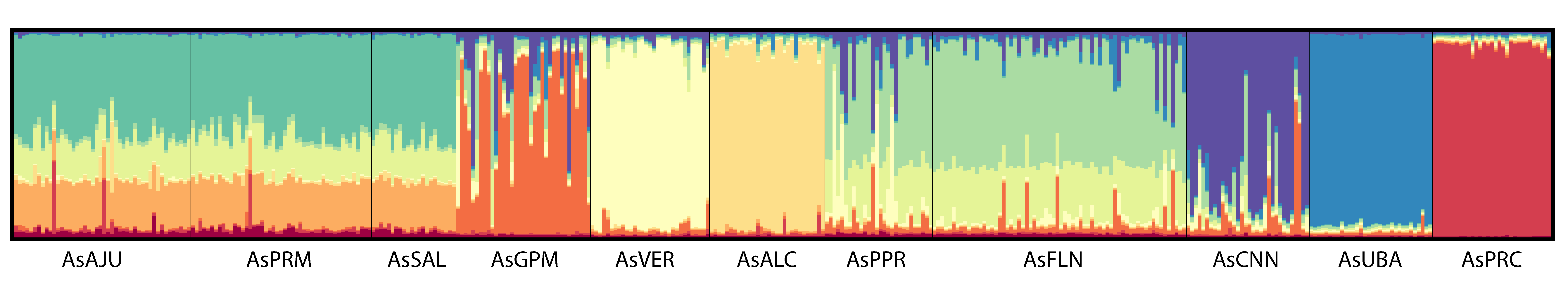

Supplement: S4 Fig — (PNG) [file pone.0118710.s008.png]

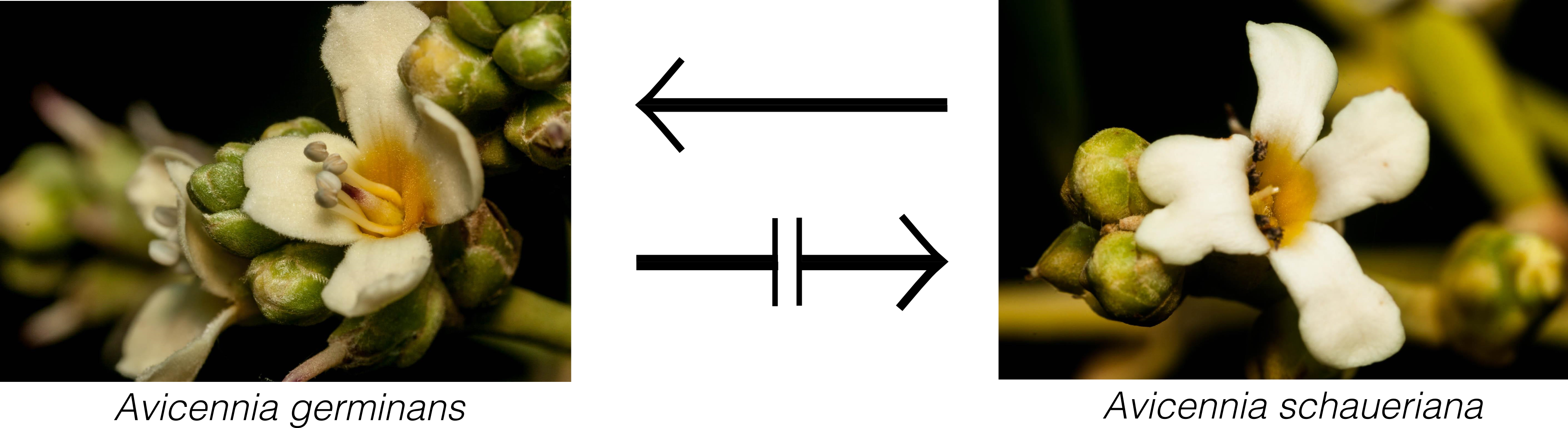

Supplement: S5 Fig — (PNG) [file pone.0118710.s009.png]
